# Urinary organophosphate metabolites, DNA methylation aging, and heart disease mortality in middle-aged and older adults: an exploratory cohort study with *in vitro* evidence of biological plausibility

**DOI:** 10.3389/fpubh.2026.1917410

**Published:** 2026-07-31

**Authors:** Yunli Song, Lihui Liang, Jing Hao, Dengfeng Ma

**Affiliations:** 1 Department of Cardiology, Taiyuan Central Hospital, Peking University First Hospital Taiyuan Hospital, Ninth Clinical Medical College of Shanxi Medical University, Taiyuan Cardiovascular Disease Research Institute, Taiyuan, China; 2 Department of Infection Control, Hongdong County People's Hospital, Linfen, China; 3 Department of Ultrasound, Hongdong County People's Hospital, Linfen, China

**Keywords:** heart disease mortality, diethylthiophosphate, DNA methylation, epigenetic clock, organophosphate pesticides

## Abstract

**Background:**

Chronic low-dose exposure to organophosphorus pesticides (OPPs) is widespread in the general population and has been linked to heart disease mortality, yet the biological pathways connecting such exposure to fatal outcomes remain poorly defined. We examined whether urinary OPP metabolites are associated with heart disease mortality and whether DNA methylation biomarkers might contribute to this association.

**Methods:**

We studied 502 adults aged ≥50 years from the National Health and Nutrition Examination Survey (NHANES) 1999–2002 cycles with urinary OPP metabolite measurements, DNA methylation profiles, and linked mortality follow-up. Survey-weighted Cox models were used to relate baseline metabolites [per 1-standard-deviation (SD) increase] to heart disease mortality, with sensitivity analyses for exposure definition and confounding. DNA methylation biomarkers that were significant after false discovery rate (FDR) correction were examined in exploratory single-mediator models, with Benjamini–Hochberg correction applied across the five indirect-effect tests, and in a secondary survival random forest. THP-1 monocytes were exposed to chlorpyrifos as a mechanistic probe of pathways suggested by the epidemiological findings.

**Results:**

Among four OPP metabolites, only diethylthiophosphate (DETP) was associated with heart disease mortality after multivariable adjustment [hazard ratio 1.33, 95% confidence interval (CI) 1.11–1.61 per 1-SD increase, FDR = 0.010], with a positive association across quartiles. After Benjamini–Hochberg correction, DETP showed FDR-significant linear associations with five DNA methylation biomarkers. In separate single-mediator models, MonoPP and ZhangAge showed nominal indirect effects at raw *p* < 0.05, with estimated proportions mediated of approximately 9.2 and 8.4%, respectively; however, neither remained statistically significant after Benjamini–Hochberg correction across the five mediation models. A survival random forest combining DETP with the five markers achieved a 5-fold cross-validated 20-year area under the curve (AUC) of 0.71 (95% CI 0.66–0.76). *In vitro*, sub-cytotoxic chlorpyrifos raised pro-inflammatory cytokines, increased DNMT1, suppressed TET2, and induced global DNA hypermethylation.

**Conclusion:**

In this exploratory analysis, higher urinary DETP was associated with greater heart disease mortality in middle-aged and older adults. MonoPP and ZhangAge showed suggestive indirect effects in separate exploratory models, each accounting for an estimated 8–9% of the association, but neither effect remained significant after FDR correction. Most of the DETP-related risk, therefore, remained unexplained by the measured methylation markers. The *in vitro* experiments provided hypothesis-generating support—most directly for inflammatory signaling—rather than confirmation of the epidemiological pathway.

## 1 Introduction

Organophosphorus pesticides (OPPs) are among the most widely used agricultural insecticides worldwide (1, 2). Their classical toxicity results from irreversible inhibition of acetylcholinesterase, resulting in the accumulation of acetylcholine and the development of acute neurotoxicity (3, 4). Farmworkers, applicators, and manufacturing workers may experience high occupational exposures that trigger cholinergic crises, but exposure in the general population differs substantially (5, 6). For people who are not occupationally exposed, exposure to OPPs is chronic and occurs at low doses, mainly through residues on conventional produce and in drinking water (7, 8).

Even after tighter limits on residential use, OPPs remain a public health concern, particularly for cardiometabolic health (3, 9). A growing body of population studies has linked urinary OPP metabolites—dialkyl phosphates that integrate exposure to several parent compounds and may also reflect preformed environmental degradation products rather than any single pesticide—to dyslipidemia, insulin resistance, and increased cardiovascular mortality (9–13). What remains unclear is how chronic, low-level exposure actually translates into fatal cardiovascular outcomes in middle-aged and older adults.

DNA methylation clocks offer one way to connect exposure with long-term risk (14, 15). Built from methylation at specific CpG sites, these markers reflect the cumulative biological effects of environmental exposures, lifestyle factors, and physiological stress (16, 17). Occupational cohorts with heavy pesticide exposure show broad methylation changes, but whether the epigenome mediates cardiovascular effects at the low levels of daily exposure seen in the general population has not been tested (18–20).

We addressed this question using the 1999–2000 and 2001–2002 cycles of the National Health and Nutrition Examination Survey (NHANES)—a period that largely predates subsequent US restrictions on residential chlorpyrifos use. We first tested whether urinary OPP metabolites were associated with heart disease mortality and then examined whether selected DNA methylation biomarkers statistically accounted for part of the observed association. Markers that were significant after false discovery rate (FDR) correction were evaluated in separate exploratory mediation models, and a survival random forest was used to characterize their joint predictive value. Finally, to examine biological plausibility, we exposed human THP-1 monocytes to chlorpyrifos—one possible lipophilic parent compound contributing to urinary diethylthiophosphate (DETP)—as a mechanistic probe rather than a one-to-one surrogate for the epidemiological biomarker.

## 2 Methods

### 2.1 Study population

NHANES is an ongoing cross-sectional program that tracks the health and nutrition of the non-institutionalized US population (21). We pooled the 1999–2000 and 2001–2002 cycles. Adults aged 50 years or older were eligible if they had urinary OPP metabolite measurements, DNA ethylation profiles, and linked mortality follow-up; 502 met these criteria. All participants gave written informed consent before enrollment (22).

Each NHANES cycle was approved by the National Center for Health Statistics (NCHS) Ethics Review Board (Protocols #98–12). The study was conducted in accordance with the Declaration of Helsinki.

### 2.2 Outcome ascertainment

Vital status and cause of death were obtained through linkage to the 2019 NHANES public-use linked mortality file, which uses a probabilistic algorithm to match survey records with National Death Index death certificates. Follow-up continued through December 31, 2019. The primary outcome was death from heart disease, defined by International Classification of Diseases codes I00–I09, I11, I13, and I20–I51 (23). Follow-up time was calculated in months from the examination date to death or censoring (maximum 249 months), and 45 heart disease deaths occurred.

### 2.3 Exposure assessment

Urinary OPP biomarkers were obtained from the Organophosphate Insecticides—Dialkyl Phosphate Metabolites—Urine files. We extracted six metabolites—dimethyl phosphate (DMP), diethyl phosphate (DEP), dimethyl thiophosphate (DMTP), diethyl thiophosphate (DETP), diethyldithiophosphate (DEDTP), and dimethyldithiophosphate (DMDTP)—and retained the four detected in at least 50% of samples (DMP, DEP, DMTP, DETP) (9). Values below the limit of detection (LOD) were set to LOD/√2. Concentrations were creatinine-adjusted (μg/g creatinine), log-transformed for normality, and standardized per 1-standard-deviation (SD) increase (24). Sampling and laboratory details are provided in the NHANES Laboratory/Medical Technician Procedures Manual.

### 2.4 DNA methylation biomarkers

DNA methylation in the 1999–2000 and 2001–2002 cycles was measured using the Illumina MethylationEPIC BeadChip v1.0 (25). From these data, we derived 17 established epigenetic markers—clocks and cell-type proportions—using published algorithms: HorvathAge, HannumAge, SkinBloodAge, PhenoAge, HorvathTelo, YangCell, ZhangAge, LinAge, WeidnerAge, VidalBraloAge, DunedinPoAm, CD8TPP, CD4TPP, Nkcell, Bcell, MonoPP, and NeuPP (26–35). Processing details—quality control, normalization, and adjustment for technical and biological covariates—are documented on the NHANES DNA Methylation Data Processing Website, and these standardized steps were used to limit batch effects. All markers were standardized per 1-SD increase before analysis.

### 2.5 Covariates

Covariates comprised age band (50–59 or ≥60 years), gender, race/ethnicity (Non-Hispanic White, Non-Hispanic Black, or Other), body mass index (BMI; <25, 25–29.9, or ≥30 kg/m^2^), alcohol use (current, former, or never), smoking (current, former, or never), hypertension, type 2 diabetes, and baseline heart disease, with the last three based on self-reported medical history. Missing covariate values (<20%) were handled by multiple imputation using chained equations (five imputed datasets, maxit = 20). Imputed variables included the baseline covariates and, in extended sensitivity models, poverty–income ratio, education, fruit and vegetable intake, total cholesterol, C-reactive protein, estimated glomerular filtration rate, physical activity, and cardiovascular- or diabetes-related medication use. Continuous variables were imputed using predictive mean matching, binary variables using logistic regression, and ordinal or nominal variables using polytomous regression. Survey-weighted Cox models were fitted within each imputed dataset, and hazard ratios (HRs), 95% confidence intervals (CIs), and *p-*values were pooled using Rubin’s rules.

### 2.6 *In vitro* cell culture and viability assay

Human THP-1 monocytes (Procell, CL-0233) were cultured in RPMI-1640 (Hyclone, SH30255.01) supplemented with 10% fetal bovine serum (Every Green, 11,011–8,611) and 1% penicillin–streptomycin (Solarbio, P1400) at 37 °C and 5% CO₂. Following an initial Cell Counting Kit-8 (CCK-8) assay (Beyotime, C0038) conducted using a chlorpyrifos (Macklin, M813721) concentration gradient, cells were treated with chlorpyrifos—one possible lipophilic parent compound contributing to urinary DETP—at sub-cytotoxic doses (10, 25, and 50 μM) in subsequent experiments.

### 2.7 Quantitative real-time PCR and global 5-mC analysis

Cells were treated with chlorpyrifos at 0, 10, 25, and 50 μM for 24 h. Total ribonucleic acid (RNA) was extracted with TRNzol Universal reagent (Tiangen, DP424) and reverse-transcribed (Tiangen, KR116). Messenger RNA (mRNA) levels of the pro-inflammatory cytokines TNF-α, IL-6, and IL-1β and the methylation-regulatory enzymes DNMT1 and TET2 were measured using quantitative real-time polymerase chain reaction (qPCR) (Vazyme, Q711) using the 2^−ΔΔCt^ method and normalized to GAPDH. Global 5-methylcytosine (5-mC) was quantified with a 5-mC enzyme-linked immunosorbent assay (ELISA) kit (Epigentek, P-1034) according to the manufacturer’s instructions.

### 2.8 Statistical analysis

Unless otherwise specified, all population analyses followed NHANES analytical guidelines for pooled 1999–2002 data and incorporated the appropriate subsample examination weights, together with sampling strata and primary sampling units, to account for the complex, stratified, multistage probability sampling design. Baseline characteristics in Table 1 were summarized as *n* (%) for categorical variables and medians (25th–75th percentiles) for continuous variables, including socioeconomic, dietary, and cardiometabolic measures and creatinine-adjusted urinary organophosphate metabolites, stratified by heart disease mortality status. Table 1 presents unweighted counts, percentages, and medians in the analytic cohort (*n* = 502) from the first completed imputed dataset; between-group *p-*values were derived from survey-weighted Wald tests.

**Table 1:** Baseline characteristics of the study population from the National Health and Nutrition Examination Survey (NHANES) 1999–2002, stratified by heart disease mortality status.

Characteristic	Level	Total (*n* = 502)	No death from heart disease (*n* = 457)	Death from heart disease (*n* = 45)	*p-*value
Age (%)	≥60 years	262 (52.2)	229 (50.1)	33 (73.3)	0.031
	50–59 years	240 (47.8)	228 (49.9)	12 (26.7)	
Gender (%)	Male	242 (48.2)	216 (47.3)	26 (57.8)	0.171
	Female	260 (51.8)	241 (52.7)	19 (42.2)	
Race/ethnicity (%)	Non-Hispanic White	225 (44.8)	199 (43.5)	26 (57.8)	0.829
	Non-Hispanic Black	111 (22.1)	101 (22.1)	10 (22.2)	
	Other	166 (33.1)	157 (34.4)	9 (20.0)	
BMI (%)	<25 kg/m^2^	142 (28.3)	127 (27.8)	15 (33.3)	0.081
	25–29.9 kg/m^2^	189 (37.6)	173 (37.9)	16 (35.6)	
	≥30 kg/m^2^	171 (34.1)	157 (34.4)	14 (31.1)	
Alcohol use (%)	Current	320 (63.7)	291 (63.7)	29 (64.4)	0.276
	Former	87 (17.3)	80 (17.5)	7 (15.6)	
	Never	95 (18.9)	86 (18.8)	9 (20.0)	
Smoking status (%)	Current	79 (15.7)	69 (15.1)	10 (22.2)	0.170
	Former	194 (38.6)	178 (38.9)	16 (35.6)	
	Never	229 (45.6)	210 (46.0)	19 (42.2)	
Hypertension (%)	No	267 (53.2)	247 (54.0)	20 (44.4)	0.151
	Yes	235 (46.8)	210 (46.0)	25 (55.6)	
Type 2 diabetes (%)	No	418 (83.3)	382 (83.6)	36 (80.0)	0.945
	Yes	84 (16.7)	75 (16.4)	9 (20.0)	
Baseline heart disease (%)	No	429 (85.5)	396 (86.7)	33 (73.3)	0.343
	Yes	73 (14.5)	61 (13.3)	12 (26.7)	
PIR (%)	<1.30	130 (25.9)	115 (25.2)	15 (33.3)	0.242
	1.30–3.49	208 (41.4)	188 (41.1)	20 (44.4)	
	≥ 3.5	164 (32.7)	154 (33.7)	10 (22.2)	
Education (%)	Less than high school	100 (19.9)	90 (19.7)	10 (22.2)	0.061
	High school/GED	207 (41.2)	184 (40.3)	23 (51.1)	
	More than high school	195 (38.8)	183 (40.0)	12 (26.7)	
Cardiovascular-related medication use (%)	No	233 (46.4)	218 (47.7)	15 (33.3)	0.102
	Yes	269 (53.6)	239 (52.3)	30 (66.7)	
Fruit and vegetable intake (g/day)	Median (IQR)	304.44 (127.88, 571.75)	305.38 (127.12, 565.62)	267.70 (133.50, 631.26)	0.883
Total cholesterol (mmol/L)	Median (IQR)	5.35 (4.71, 6.08)	5.38 (4.71, 6.10)	5.28 (4.50, 6.00)	0.529
C-reactive protein (mg/dL)	Median (IQR)	0.26 (0.12, 0.61)	0.26 (0.12, 0.62)	0.24 (0.13, 0.47)	0.732
eGFR (mL/min/1.73 m^2^)	Median (IQR)	87.50 (70.07, 102.13)	88.33 (72.17, 102.81)	73.19 (62.11, 91.35)	0.735
Physical activity (MET-min/week)	Median (IQR)	0.00 (0.00, 855.75)	0.00 (0.00, 931.00)	0.00 (0.00, 720.42)	0.023
Creatinine-adjusted urinary OPP metabolites (μg/g creatinine)					
DMP	Median (IQR)	1.14 (0.38, 4.05)	1.21 (0.39, 4.28)	0.83 (0.32, 2.46)	0.072
DEP	Median (IQR)	0.90 (0.21, 3.13)	0.88 (0.21, 3.10)	1.11 (0.24, 3.42)	0.025
DMTP	Median (IQR)	1.22 (0.33, 6.62)	1.24 (0.33, 6.98)	0.94 (0.38, 4.60)	0.037
DETP	Median (IQR)	0.72 (0.15, 1.75)	0.65 (0.14, 1.75)	1.05 (0.61, 1.65)	0.166

Baseline characteristics are presented for 502 participants aged ≥50 years. Categorical variables are shown as unweighted counts followed by percentages in parentheses. Continuous variables are shown as medians with the 25th and 75th percentiles in parentheses. Between-group comparisons used design-adjusted survey-weighted Wald tests with NHANES examination weights, sampling strata, and primary sampling units. PIR, poverty–income ratio; BMI, body mass index; eGFR, estimated glomerular filtration rate; DMP, dimethyl phosphate; DEP, diethyl phosphate; DMTP, dimethyl thiophosphate; DETP, diethyl thiophosphate; GED, General Educational Development; IQR, interquartile range; MET, metabolic equivalent of task; OPP, organophosphorus pesticide.

Survey-weighted Cox proportional hazards models related each creatinine-adjusted urinary metabolite (per 1-SD increase) to heart disease mortality, with adjustment for age band (50–59 or ≥60 years), gender, race/ethnicity, BMI category, alcohol use, smoking, hypertension, type 2 diabetes, and baseline heart disease. Benjamini–Hochberg false discovery rate (FDR) correction was applied across the four continuous metabolites, and the proportional-hazards assumption was checked using Schoenfeld residuals (all *p* > 0.05). The metabolite that remained associated with mortality was additionally modeled in quartiles, with the lowest quartile as the reference.

Several sensitivity analyses tested the robustness of the DETP–mortality association: (i) alternative approaches to urine-dilution adjustment (creatinine as a denominator versus creatinine included as a model covariate); (ii) sequential addition of socioeconomic, dietary, cardiometabolic, and medication covariates—poverty–income ratio, education, fruit and vegetable intake, total cholesterol, C-reactive protein, estimated glomerular filtration rate, physical activity, and cardiovascular- or diabetes-related medication use; (iii) exclusion of participants with baseline heart disease; and (iv) a Fine–Gray subdistribution hazards model treating death from causes other than heart disease as the competing event. This model did not incorporate the NHANES survey design and was therefore considered an exploratory sensitivity analysis.

The association between DETP and DNA methylation was assessed in two steps. First, survey-weighted linear regression related DETP (per 1-SD increase) to each of the 17 methylation markers using the same covariates, with Benjamini–Hochberg FDR correction across the 17 markers. Second, the markers that survived correction (FDR < 0.05) were carried forward into survey-weighted Cox models for heart disease mortality, with FDR correction applied across these five mortality models.

The mediation analysis was conducted using the first completed imputed dataset and did not incorporate the NHANES survey design. The survival random forest was also fitted using the first completed imputed dataset; examination weights were applied as case weights during model fitting, but sampling strata and primary sampling units were not modeled. Accordingly, both analyses were treated as secondary and were not interpreted as fully nationally representative. Each FDR-significant marker was evaluated in a separate single-mediator model of the DETP–mortality association using Cox-based mediation analysis implemented in the CMAverse package. The natural indirect effect (NIE), natural direct effect (NDE), total effect (TE), and proportion mediated (PM) were estimated with 95% CIs. Because five single-mediator models were examined, Benjamini–Hochberg correction was additionally applied across the five NIE *p-*values. Raw *p-*values are reported to describe exploratory signals, whereas FDR-adjusted *p-*values were used to assess multiplicity-controlled statistical significance. Because the epigenetic clocks are mutually correlated, indirect effects from separate single-mediator models should not be interpreted as independent, additive pathways, and the small number of events (45 heart disease deaths) further limited statistical power.

As a secondary analysis, a survival random forest (ranger package) combined DETP with the five FDR-significant markers, with examination weights applied as case weights. Discrimination was quantified using the 20-year time-dependent area under the curve (AUC) calculated from out-of-fold predictions generated by 5-fold cross-validation. A 95% CI was estimated from 1,000 bootstrap resamples of the out-of-fold predictions using the percentile method, and variable importance was ranked using Shapley additive explanation (SHAP) values (fastshap package). Given only 45 events and six predictors, the cross-validated discrimination and importance rankings should be regarded as unstable.

For the *in vitro* experiments (four biological replicates per condition), relative mRNA expression was calculated using the 2^−ΔΔCt^ method and normalized to GAPDH, and global 5-mC was expressed relative to the vehicle control; data are presented as the mean ± SD. Each chlorpyrifos concentration (10, 25, and 50 μM) was compared with the vehicle control (0 μM) by one-way analysis of variance (ANOVA) followed by Dunnett’s *post-hoc* test.

Analyses used R version 4.4.1, with two-sided *p* < 0.05 as the significance threshold.

## 3 Results

### 3.1 Baseline characteristics of the study population

Table 1 summarizes the 502 adults aged ≥50 years included in the analysis, together with their socioeconomic, dietary, and cardiometabolic characteristics and urinary organophosphate metabolite concentrations. Participants who subsequently died of heart disease were, at baseline, more likely to be aged ≥60 years (73.3% vs. 50.1%, *p* = 0.031). Gender, race/ethnicity, BMI category, alcohol use, smoking, hypertension, type 2 diabetes, and baseline heart disease were broadly comparable between groups. Physical activity, DEP, and DMTP also differed between groups at the nominal *p* < 0.05 level; however, these comparisons were unadjusted for multiple testing, and neither DEP nor DMTP was associated with mortality in the adjusted Cox models.

### 3.2 Urinary DETP, but not other metabolites, is associated with heart disease mortality

**Figure 1:**
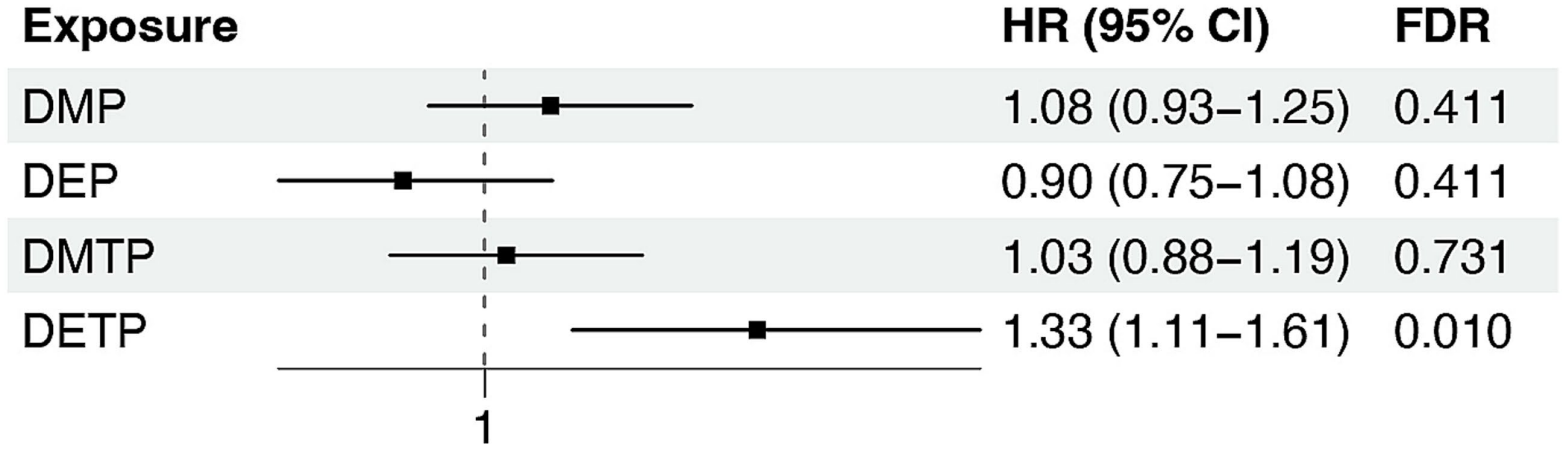
Associations between urinary organophosphorus pesticide (OPP) metabolites and heart disease mortality. Hazard ratios (*HRs*) and 95% confidence intervals (*CIs*) were estimated using survey-weighted Cox proportional hazards regression models. *p-*values are false discovery rate (*FDR*)-adjusted across the four continuous metabolites. *HRs* are expressed per 1-standard deviation (*SD*) increase in log-transformed biomarker levels. The models were adjusted for age band, gender, race/ethnicity, body mass index (*BMI*), alcohol use, smoking status, hypertension, type 2 diabetes, and baseline heart disease. Urinary OPP metabolite concentrations (DMP, DEP, DMTP, and DETP) were creatinine-adjusted, log-transformed, and converted to *z*-scores prior to analysis. Survey-weighted Cox models incorporated the National Health and Nutrition Examination Survey (NHANES) complex sampling design. DMP, dimethyl phosphate; DEP, diethyl phosphate; DMTP, dimethyl thiophosphate; DETP, diethyl thiophosphate.

**Figure 2:**
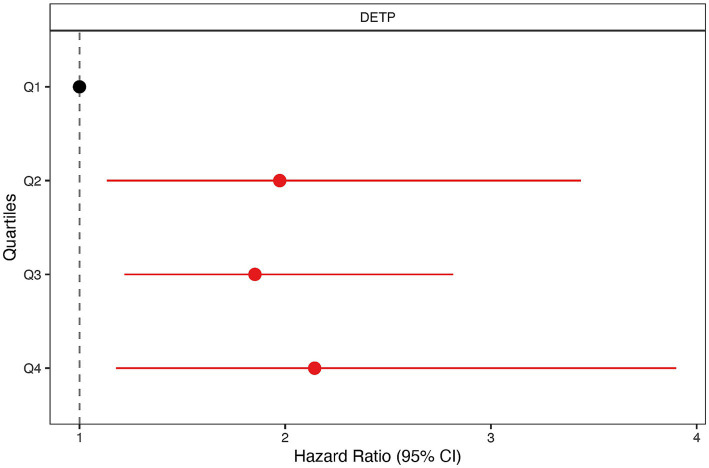
Quartile-based associations between urinary organophosphorus pesticide (OPP) metabolites and heart disease mortality. The forest plot displays hazard ratios (HRs) and 95% confidence intervals (CIs) for quartiles of DMP, DEP, DMTP, and DETP, with the lowest quartile (Q1) serving as the reference group. Models were fully adjusted for covariates, including age band, gender, race/ethnicity, body mass index (BMI), alcohol use, smoking status, hypertension, type 2 diabetes, and baseline heart disease. Survey-weighted Cox models were used. DMP, dimethyl phosphate; DEP, diethyl phosphate; DMTP, dimethyl thiophosphate; DETP, diethyl thiophosphate.

We first examined which urinary organophosphate metabolite was associated with heart disease mortality. In survey-weighted Cox proportional hazards models, DETP was the only metabolite that showed a significant association (HR 1.33 per 1-SD increase, 95% CI 1.11–1.61, FDR = 0.010; Figure 1), and the proportional-hazards assumption was satisfied (*p* > 0.05). This adjusted association is directionally consistent with the higher baseline DETP among decedents shown in Table 1, which did not reach significance in the unadjusted survey-weighted comparison (*p* = 0.166). DMP, DEP, and DMTP were not significantly associated with mortality. The DETP association was positive across quartiles (Figure 2): relative to the lowest quartile (Q1), the HRs were 1.97 (95% CI 1.13–3.44) for Q2, 1.85 (95% CI 1.22–2.82) for Q3, and 2.14 (95% CI 1.18–3.90) for Q4, although the limited number of heart disease deaths per quartile made these estimates imprecise. DETP was therefore carried forward as the exposure of interest in all subsequent analyses.

Having identified DETP as the exposure of interest, we next asked whether its association with mortality was robust to alternative exposure definitions and to more extensive adjustment for diet, socioeconomic status, cardiometabolic factors, and competing mortality (Table 2). The survey-weighted association persisted across creatinine-adjustment approaches (HRs 1.26–1.33 per 1-SD increase), strengthened modestly with sequential confounder adjustment (HR up to 1.44, 95% CI 1.16–1.78), and remained after excluding participants with baseline heart disease (*n* = 429; 33 deaths; HR 1.34, 95% CI 1.10–1.63). An unweighted Fine–Gray model treating death from causes other than heart disease as the competing event yielded a directionally consistent but imprecise estimate that included the null (subdistribution HR 1.29, 95% CI 0.98–1.69). Because this model did not incorporate the NHANES survey design, it was interpreted only as an exploratory sensitivity analysis. We next examined the association between DETP and DNA methylation biomarkers.

**Table 2:** Sensitivity analyses of the association between urinary diethylthiophosphate [DETP; per 1-standard-deviation (SD) increase] and heart disease mortality.

Section	Analysis	Exposure	*N*	Events	HR (95% CI)	*p-*value
A. Primary and creatinine sensitivity	Primary model (survey-weighted Cox)	DETP	502	45	1.33 (1.11–1.61)	0.0025
A. Primary and creatinine sensitivity	Uncorrected DETP + urinary creatinine	DETP	502	45	1.26 (1.03–1.54)	0.0225
A. Primary and creatinine sensitivity	Uncorrected DETP only	DETP	502	45	1.26 (1.04–1.52)	0.0182
B. Sequential confounder adjustment	Model 0: Main covariates	DETP	502	45	1.33 (1.11–1.61)	0.0025
B. Sequential confounder adjustment	Model 1: + PIR, education	DETP	502	45	1.35 (1.12–1.64)	0.0021
B. Sequential confounder adjustment	Model 2: + fruit/vegetable intake	DETP	502	45	1.38 (1.14–1.68)	0.0012
B. Sequential confounder adjustment	Model 3: + cholesterol, CRP, eGFR	DETP	502	45	1.44 (1.16–1.78)	<0.001
B. Sequential confounder adjustment	Model 4: + physical activity	DETP	502	45	1.43 (1.16–1.75)	<0.001
B. Sequential confounder adjustment	Model 5: + medications	DETP	502	45	1.42 (1.16–1.74)	<0.001
C. Exclude baseline heart disease	All participants	DETP	502	45	1.33 (1.11–1.61)	0.0025
C. Exclude baseline heart disease	Excluding baseline heart disease	DETP	429	33	1.34 (1.10–1.63)	0.0032
D. Competing-risk analysis (Fine–Gray; unweighted)	Fine–Gray subdistribution hazards model	DETP	502	45	1.29 (0.98–1.69)	0.0660

Unless otherwise noted, HRs and 95% CIs were estimated from survey-weighted Cox proportional hazards models adjusted for age band, gender, race/ethnicity, BMI category, alcohol use, smoking, hypertension, type 2 diabetes, and baseline heart disease. Section A compares the main creatinine-adjusted DETP definition with models using uncorrected DETP, with or without urinary creatinine as a covariate. Section B sequentially adds the poverty–income ratio, education, fruit and vegetable intake, total cholesterol, C-reactive protein, estimated glomerular filtration rate, physical activity, and cardiovascular- or diabetes-related medication use. Section C excludes participants with baseline heart disease. Section D reports an unweighted Fine–Gray subdistribution HR for death from heart disease, treating death from causes other than heart disease as the competing event. Because the Fine–Gray model did not incorporate the NHANES complex survey design, it was considered an exploratory sensitivity analysis. HR, hazard ratio; CI, confidence interval; BMI, body mass index; CRP, C-reactive protein; eGFR, estimated glomerular filtration rate; N, analytic sample size; NHANES, National Health and Nutrition Examination Survey; PIR, poverty–income ratio.

### 3.3 DETP is associated with monocyte proportion and epigenetic age markers

**Figure 3:**
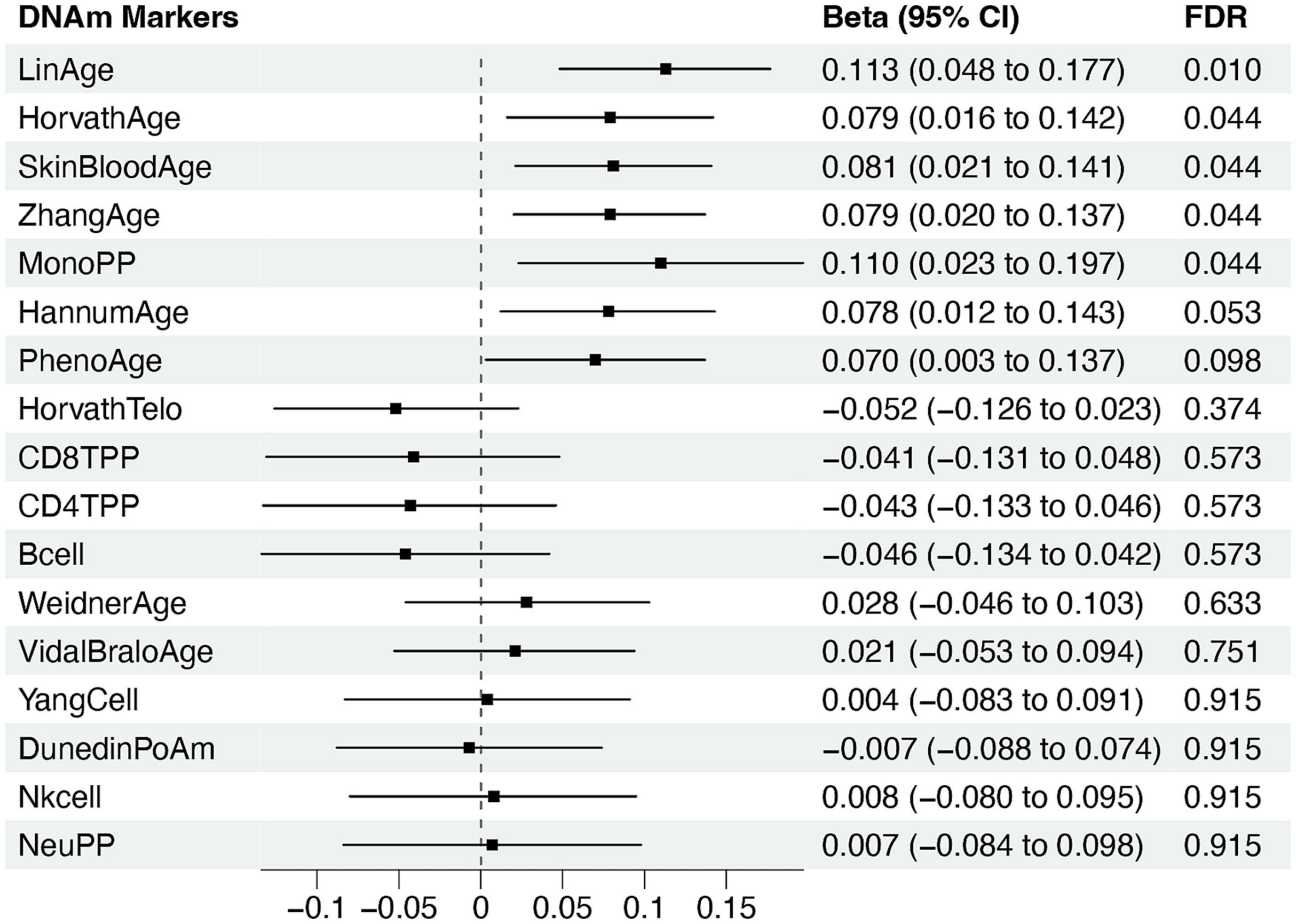
Associations between baseline urinary diethylthiophosphate (DETP) concentrations and DNA methylation biomarkers. The forest plot displays the β coefficients and 95% confidence intervals (CIs) from survey-weighted linear regression models assessing the relationship between *z*-score-transformed urinary DETP and various epigenetic biomarkers. Models were fully adjusted for covariates, including age band, gender, race/ethnicity, body mass index (BMI), alcohol use, smoking status, hypertension, type 2 diabetes, and baseline heart disease. Benjamini–Hochberg false discovery rate (FDR) correction was applied across the 17 markers. Identical FDR values for several markers can occur when raw *p*-values are closely ranked.

We then evaluated the association between DETP and the epigenetic markers. In survey-weighted linear models, DETP (per 1-SD increase) was positively associated, after FDR correction, with five of the 17 methylation markers: LinAge (*β* = 0.113, FDR = 0.011), MonoPP (*β* = 0.110, FDR = 0.044), SkinBloodAge (*β* = 0.081, FDR = 0.044), HorvathAge (*β* = 0.079, FDR = 0.044), and ZhangAge (*β* = 0.079, FDR = 0.044) (Figure 3). The remaining 12 markers did not survive correction. The DETP-responsive set thus spanned both methylation-based monocyte proportion and multi-tissue epigenetic age.

### 3.4 DETP-responsive methylation markers are associated with heart disease mortality

**Figure 4:**
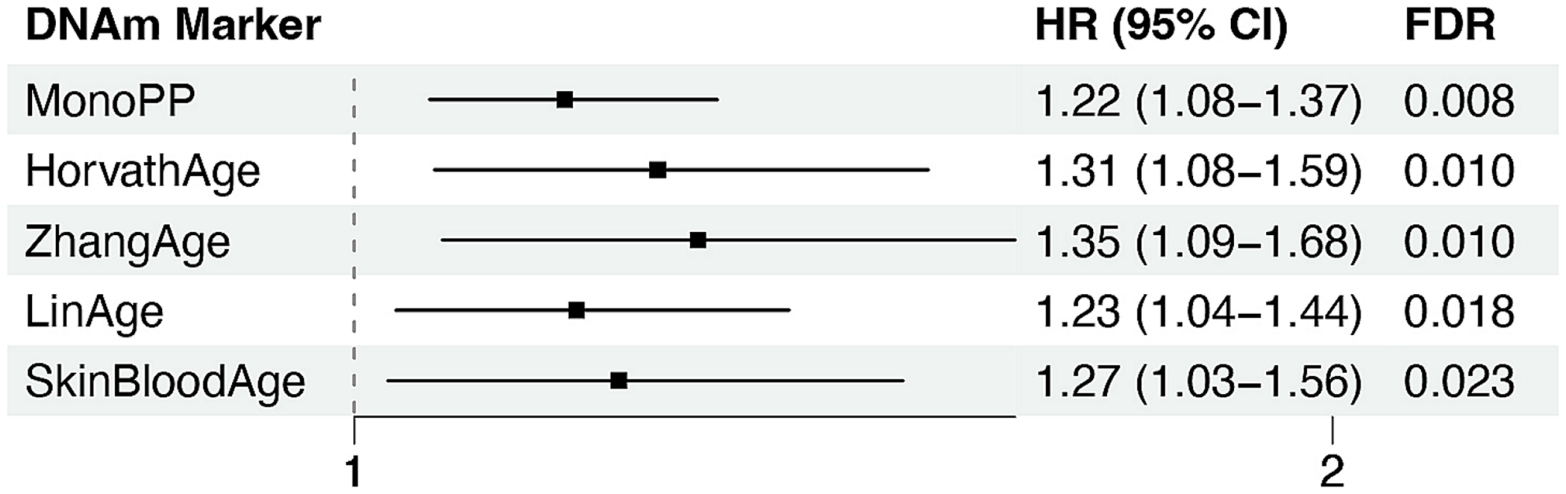
Associations between diethylthiophosphate (DETP)-related DNA methylation biomarkers and heart disease mortality. The forest plot presents results from survey-weighted Cox proportional hazards models evaluating the prospective associations of five DNA methylation biomarkers that were significant after false discovery rate (FDR) correction [LinAge, MonoPP, SkinBloodAge, HorvathAge, and ZhangAge; per 1-standard-deviation (SD) increase] with heart disease mortality. Models were fully adjusted for covariates, including age band, gender, race/ethnicity, body mass index (BMI), alcohol use, smoking status, hypertension, type 2 diabetes, and baseline heart disease.

We next examined whether these DETP-responsive markers were also associated with heart disease mortality before carrying them forward to the exploratory mediation analysis. In survey-weighted Cox models, all five were associated with heart disease mortality (Figure 4): LinAge (HR 1.23, 95% CI 1.04–1.44; FDR = 0.018), MonoPP (HR 1.22, 95% CI 1.08–1.37; FDR = 0.008), SkinBloodAge (HR 1.27, 95% CI 1.03–1.56; FDR = 0.023), HorvathAge (HR 1.31, 95% CI 1.08–1.59; FDR = 0.009), and ZhangAge (HR 1.35, 95% CI 1.09–1.68; FDR = 0.009). All five markers were associated with both DETP and heart disease mortality and were therefore carried forward to the exploratory mediation analysis.

### 3.5 Exploratory mediation analysis of the DETP–mortality association

**Figure 5:**
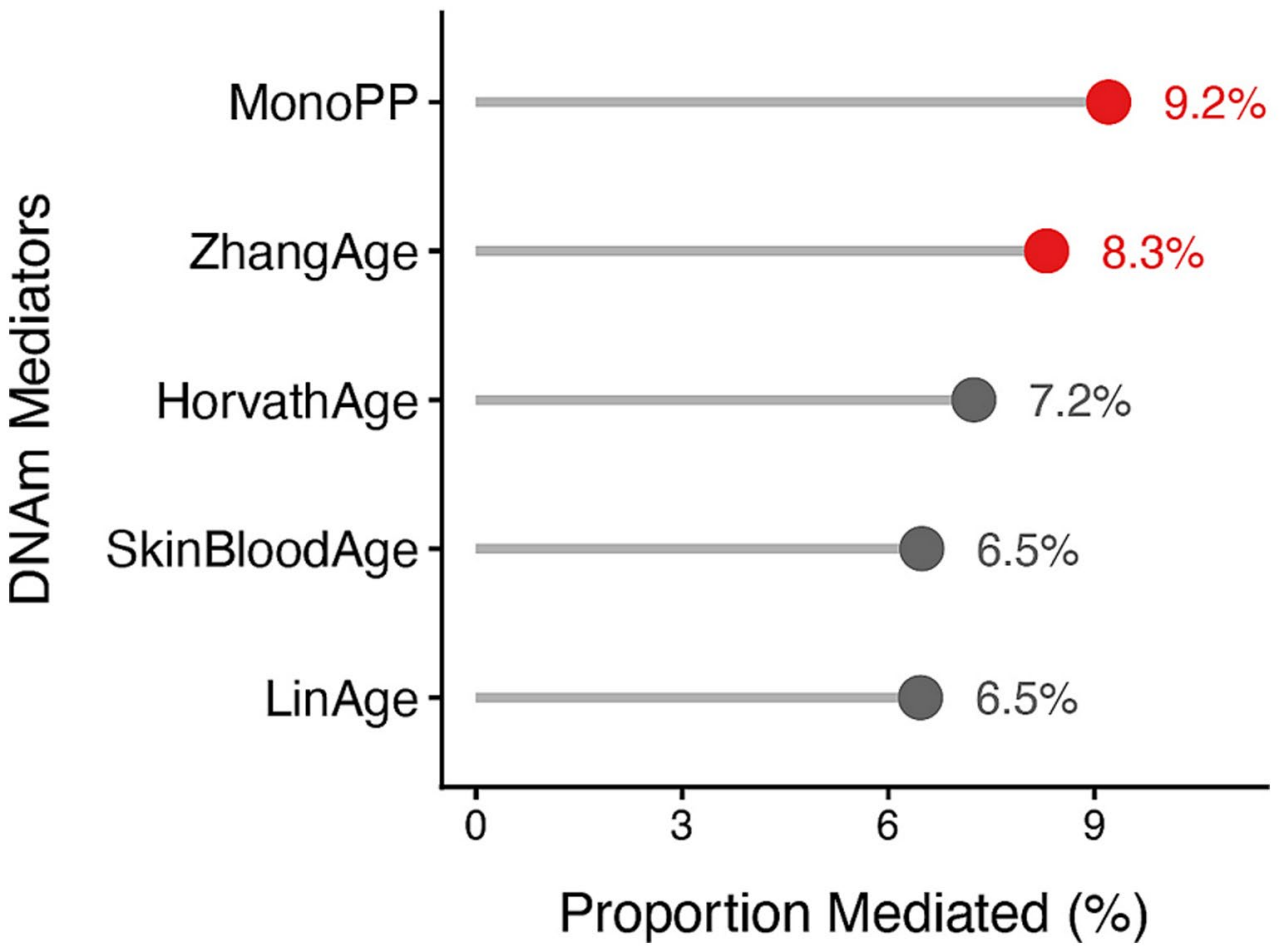
Lollipop chart illustrating the proportion of the diethylthiophosphate (DETP)-heart disease mortality association mediated by DNA methylation biomarkers. The *x*-axis represents the proportion mediated (PM), and the *y*-axis lists candidate DNA methylation biomarkers evaluated in the mediation analysis. Colored markers indicate nominal indirect effects at raw *p* < 0.05 for MonoPP and ZhangAge. Neither indirect effect remained statistically significant after Benjamini–Hochberg correction across the five mediation models.

To explore whether the selected methylation markers statistically accounted for part of the observed DETP–mortality association, we performed separate single-mediator analyses using CMAverse (Table 3 and Figure 5). MonoPP and ZhangAge showed nominal indirect effects at raw *p* < 0.05. The estimated proportion mediated was 9.2% for MonoPP (95% CI 0.012–0.206; NIE raw *p* = 0.016) and 8.4% for ZhangAge (95% CI 0.002–0.235; NIE raw *p* = 0.040). HorvathAge showed a similar point estimate but did not reach the nominal threshold (raw *p* = 0.072), whereas SkinBloodAge (raw *p* = 0.104) and LinAge (raw *p* = 0.188) showed weaker evidence of an indirect effect. After Benjamini–Hochberg correction across the five mediation models, none of the indirect effects remained statistically significant (FDR = 0.080 for MonoPP, 0.100 for ZhangAge, 0.120 for HorvathAge, 0.130 for SkinBloodAge, and 0.188 for LinAge). These findings therefore represent suggestive, hypothesis-generating signals rather than multiplicity-controlled evidence of mediation.

**Table 3:** Survival mediation analysis of the association between urinary diethylthiophosphate (DETP) and heart disease mortality through DNA methylation biomarkers.

Mediator	NIE		NDE		TE		PM	
	HR (95% CI)	*p*-value	HR (95% CI)	*p*-value	HR (95% CI)	*p*-value	PM (95% CI)	*p*-value
LinAge	1.01 (0.99–1.05)	0.188	1.23 (1.11–1.39)	0.002	1.25 (1.12–1.42)	<0.001	0.064 (−0.030 to 0.24)	0.188
MonoPP	1.02 (1.00–1.05)	0.016	1.26 (1.14–1.42)	<0.001	1.28 (1.16–1.44)	<0.001	0.092 (0.012–0.21)	0.016
SkinBloodAge	1.01 (0.99–1.04)	0.104	1.24 (1.13–1.41)	<0.001	1.26 (1.14–1.43)	<0.001	0.066 (−0.013 to 0.18)	0.104
HorvathAge	1.02 (0.99–1.04)	0.072	1.24 (1.11–1.43)	<0.001	1.25 (1.12–1.45)	<0.001	0.074 (−0.004 to 0.21)	0.072
ZhangAge	1.02 (1.00–1.05)	0.040	1.24 (1.12–1.40)	<0.001	1.26 (1.14–1.43)	<0.001	0.084 (0.002–0.24)	0.040

Natural indirect effects, natural direct effects, total effects, and proportions mediated were estimated in separate single-mediator models. Raw *p*-values are presented in the table. Benjamini–Hochberg-adjusted *p*-values for the five NIE tests were 0.188 for LinAge, 0.080 for MonoPP, 0.130 for SkinBloodAge, 0.120 for HorvathAge, and 0.100 for ZhangAge. Thus, MonoPP and ZhangAge showed nominal indirect effects at raw *p* < 0.05, but no indirect effect remained statistically significant at FDR < 0.05. NIE, natural indirect effect; NDE, natural direct effect; TE, total effect; PM, proportion mediated; HR, hazard ratio; CI, confidence interval; FDR, false discovery rate.

### 3.6 Survival random forest prediction

**Figure 6:**
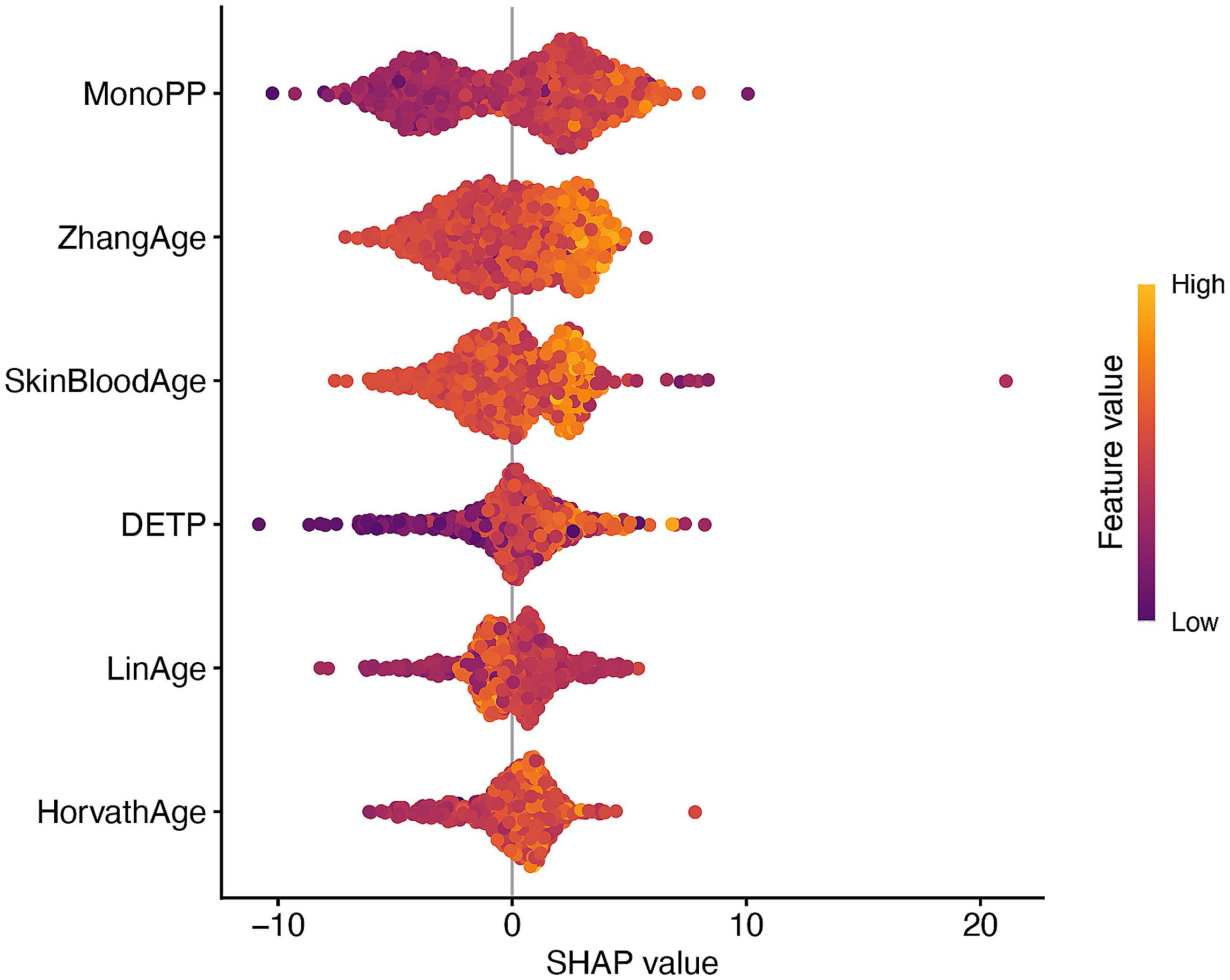
Shapley additive explanations (SHAP) summary beeswarm plot ranking variable importance for the 20-year prediction of heart disease mortality. Variable importance was derived from a survival random forest model incorporating diethylthiophosphate (DETP) and five core DNA methylation biomarkers. SHAP values reflect predictive contribution rather than causal or mediating importance. Variables are ranked on the *y*-axis in descending order of their global importance (ZhangAge, SkinBloodAge, MonoPP, HorvathAge, DETP, and LinAge). Each dot represents a single participant; the color gradient indicates the original value of the feature (red for high and blue for low), and the position on the *x*-axis shows the SHAP value’s impact on the model’s output (mortality risk).

As an exploratory secondary analysis, we combined DETP with the five markers in a survival random forest to assess their joint predictive value. The model achieved a 20-year cross-validated AUC of 0.71 (bootstrap 95% CI 0.66–0.76). SHAP-based attribution ranked the features in the order ZhangAge, SkinBloodAge, MonoPP, HorvathAge, DETP, and LinAge (Figure 6), placing several epigenetic-age and monocyte-composition markers above DETP and partially overlapping with the markers showing nominal indirect-effect signals (MonoPP and ZhangAge); these rankings, however, reflect predictive rather than causal importance. Overall, DETP and the five markers together provided moderate cross-validated discrimination, which should be interpreted cautiously given the small number of events.

### 3.7 Chlorpyrifos induces inflammatory and methylation-related transcriptional changes in THP-1 cells

The population analysis implicated monocyte-related inflammation and disturbed methylation control as plausible downstream processes. To test whether organophosphate exposure could elicit such responses experimentally, we exposed THP-1 monocytes to chlorpyrifos—one possible lipophilic parent compound contributing to urinary DETP, which is not specific to chlorpyrifos and may also reflect other parent compounds or preformed environmental metabolites.

**Figure 7:**
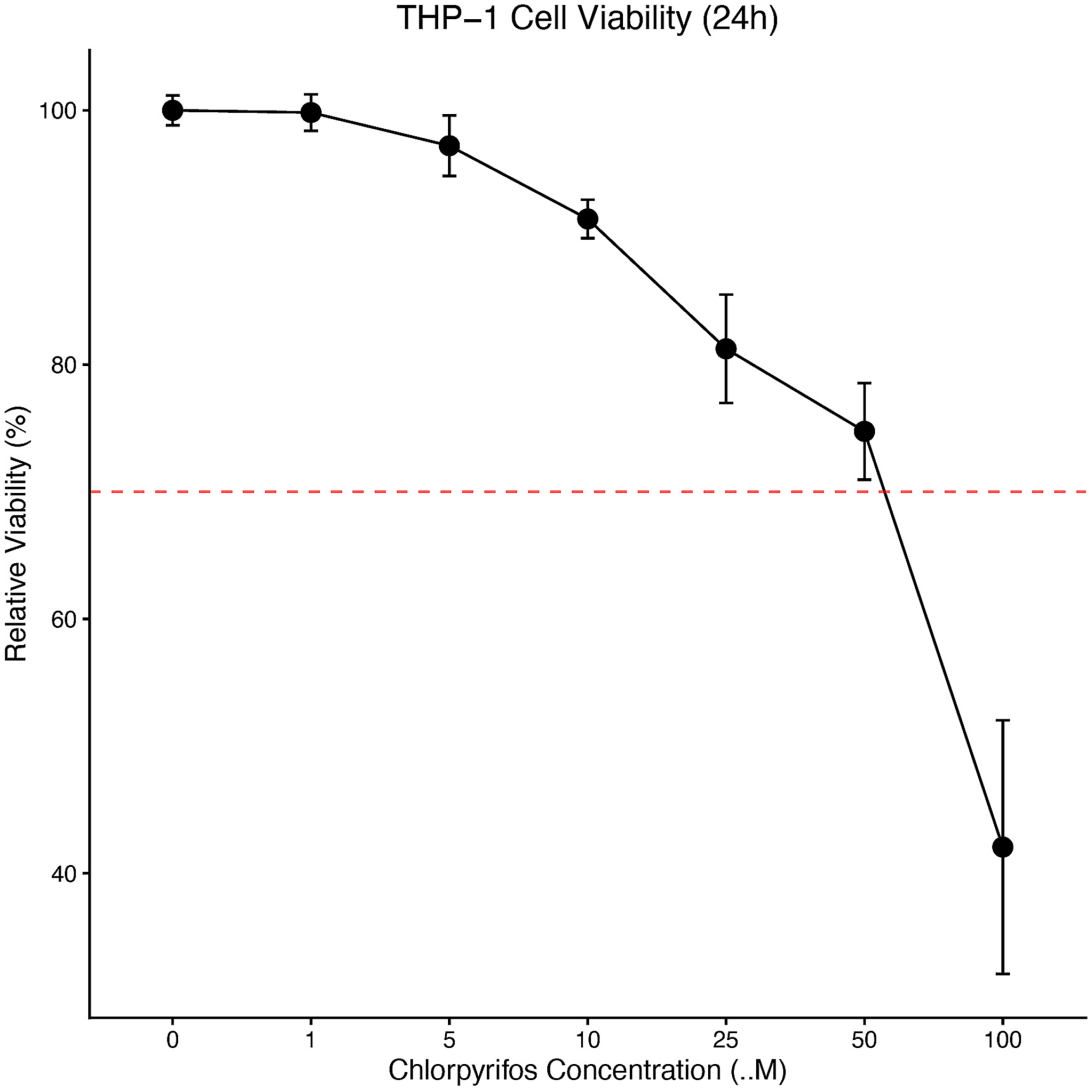
Cell viability of human THP-1 monocytes exposed to chlorpyrifos. Relative cell viability was assessed by the Cell Counting Kit-8 (CCK-8) assay after 24 h of exposure to a chlorpyrifos concentration gradient (0–100 μM). The dashed line indicates the 70% viability threshold. Data are presented as the mean ± standard deviation (SD) of four biological replicates.

We first established a sub-cytotoxic dose window using the CCK-8 assay. Cell viability remained above 70% at concentrations up to 50 μM (74.8% at 50 μM) but fell sharply to 42.1% at 100 μM (Figure 7); concentrations of 10, 25, and 50 μM were therefore used for all subsequent readouts. These concentrations exceed typical environmental exposure levels and were selected to remain sub-cytotoxic while eliciting measurable pathway responses for hypothesis generation.

**Figure 8:**
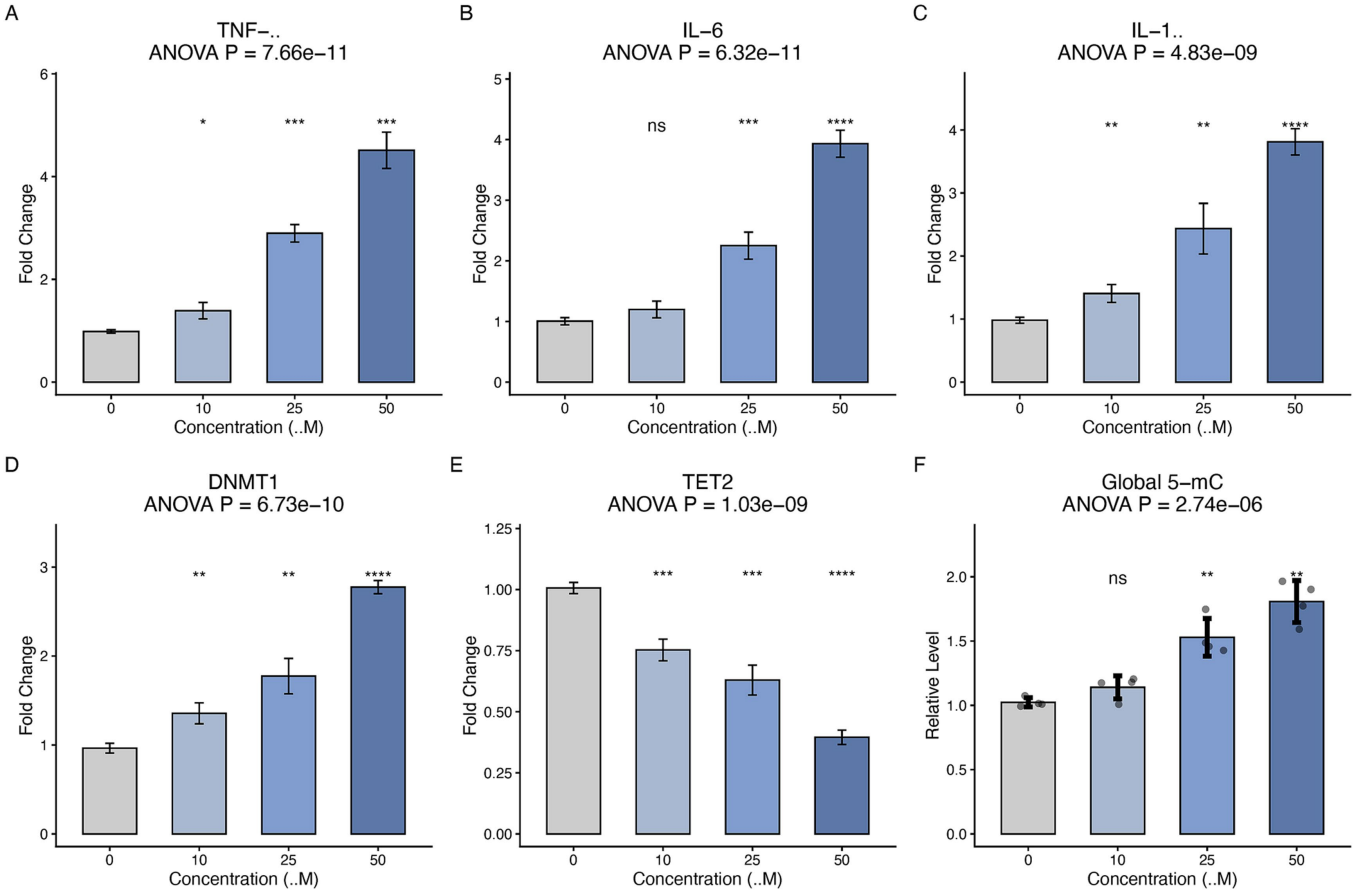
Chlorpyrifos-induced inflammatory and methylation-related responses in THP-1 monocytes. THP-1 monocytes were exposed to sub-cytotoxic concentrations of chlorpyrifos (0, 10, 25, and 50 μM) for 24 h. **(A)** TNF-α, **(B)** IL-6, and **(C)** IL-1β: dose-dependent upregulation of pro-inflammatory cytokine mRNA quantified by quantitative real-time polymerase chain reaction (qPCR). **(D)** DNMT1 and **(E)** TET2: expression of DNA methylation-regulatory enzymes quantified by qPCR. **(F)** Global 5-methylcytosine (5-mC) levels quantified by enzyme-linked immunosorbent assay (ELISA). Data are presented as the mean ± standard deviation (SD) (*n* = 4). *p < 0.05, **p < 0.01, ***p < 0.001, and ****p < 0.0001 compared with the vehicle control (0 μM), as evaluated by one-way analysis of variance (ANOVA) with Dunnett’s *post-hoc* test (multiplicity-controlled comparisons vs. 0 μM).

We then examined the inflammatory response. Chlorpyrifos increased pro-inflammatory cytokine mRNA in a dose-dependent manner, consistent with the monocyte-related signal implied by MonoPP: at 50 μM, expression rose relative to the vehicle control for TNF-α (4.51 ± 0.35 vs. 0.99 ± 0.04, *p* < 0.001), IL-6 (3.93 ± 0.22 vs. 1.00 ± 0.06, *p* < 0.001), and IL-1β (3.81 ± 0.21 vs. 0.98 ± 0.05, *p* < 0.001) (Figure 8).

We next asked whether the methylation-related changes occurred in parallel. Chlorpyrifos also perturbed methylation-regulatory gene expression, increasing DNMT1 (2.78 ± 0.07 vs. 0.96 ± 0.06, *p* < 0.001 at 50 μM) while decreasing the demethylase TET2 (0.40 ± 0.03 vs. 1.01 ± 0.02, *p* < 0.001 at 50 μM) (Figure 8). The net effect was global hypermethylation, with 5-mC reaching 1.81 ± 0.16-fold over control at 50 μM (Dunnett’s test, *p* < 0.001) (Figure 8). These cellular responses are consistent with the inflammatory and methylation-related pathways suggested by the observational analysis, but they do not validate any specific epidemiological mediator or epigenetic clock.

## 4 Discussion

In this exploratory cohort of 502 middle-aged and older adults from NHANES 1999–2002, higher urinary DETP was associated with heart disease mortality during long-term follow-up. In separate single-mediator models, MonoPP and ZhangAge showed nominal indirect effects at raw *p* < 0.05, with estimated proportions mediated of approximately 8–9%; however, none of the five indirect effects remained significant after FDR correction, and most of the excess risk remained unexplained. In a secondary survival random forest, DETP and the five FDR-significant methylation markers yielded a cross-validated 20-year AUC of 0.71 (95% CI 0.66–0.76), a result that requires external validation given that only 45 events occurred.

These findings are consistent with a possible continuum of OPP-related cardiovascular effects. Acute, high-dose exposure causes immediate neurotoxicity, whereas in our observational analysis, higher urinary DETP was associated with greater long-term heart disease mortality. DETP is a nonspecific dialkyl phosphate metabolite that may reflect exposure to several lipophilic parent compounds, including chlorpyrifos and diazinon, as well as preformed environmental degradation products (36, 37). The positive association observed across the upper exposure quartiles suggests possible vulnerability of the aging cardiovascular system to these compounds (38). Because these NHANES cycles largely predate major US residential chlorpyrifos restrictions, extrapolation to current exposure patterns warrants caution.

Identifying DNA methylation markers associated with both exposure and outcome provides a plausible, though exploratory, biological context for the OPP–cardiovascular link. The *in vitro* findings provide complementary support for the biological plausibility of inflammatory signaling, whereas the epigenetic-aging findings remain hypothesis-generating. The MonoPP signal warrants further consideration. Monocytes drive early atherogenesis through endothelial infiltration and foam-cell formation (39, 40). In our model, chlorpyrifos increased transcription of the pro-inflammatory cytokines TNF-α, IL-6, and IL-1β in THP-1 cells, broadly consistent with the monocyte-related signal associated with MonoPP. A methylation-based skew toward monocytes could therefore reflect a chronic inflammatory shift that destabilizes plaques (41–43), although MonoPP captures inferred cell composition rather than direct monocyte activation. Among the epigenetic clocks, ZhangAge showed a nominal indirect effect at raw *p* < 0.05, although this association did not remain significant after FDR correction. Associations involving ZhangAge, HorvathAge, and the other clocks may partly reflect general biological aging, frailty, or comorbidity rather than organophosphate-specific biology (44–46). Consistent with an epigenetic mechanism, chlorpyrifos disturbed the methylation machinery *in vitro*, increasing DNMT1 expression, decreasing TET2 expression, and increasing global 5-mC (47), in line with DNMT1-linked epigenetic regulation (48). DNMT1-dependent methylation has also been implicated in immune-cell senescence in cardiac settings (49). Two caveats apply: epigenetic clocks are based on locus-specific CpG patterns rather than global methylation, and a short cellular exposure cannot reproduce the slow dynamics of biological aging.

The survival random forest and SHAP analysis provided an exploratory complement to the primary Cox models. In environmental epidemiology, standard semi-parametric models may not fully capture non-linear dose–response patterns or collinearity among correlated clocks (50). The forest reached a cross-validated 20-year AUC of 0.71 (95% CI 0.66–0.76), broadly consistent with prior reports on methylation-based mortality prediction in older adults (51, 52), although estimates are likely unstable with only 45 events. SHAP rankings placed ZhangAge, SkinBloodAge, MonoPP, and HorvathAge higher than baseline DETP; however, these rankings reflect predictive contribution rather than causal or mediating importance, and with correlated clocks, importance can be redistributed across features in small samples (19, 53).

These interpretations have important limitations. First, the modest sample size (*n* = 502) and the small number of heart disease deaths (*n* = 45) limit power for multivariable Cox models, mediation, and machine learning, so predictive performance and importance rankings need external validation in larger cohorts. Second, exposure assessment was based on a single baseline spot urine measurement; given the short half-lives of dialkyl phosphates, such a measurement chiefly reflects recent rather than cumulative exposure over follow-up (54), although stable dietary habits in middle-aged and older adults may allow a baseline value to approximate longer-term intake. Third, heart disease mortality was ascertained from death certificates and may be misclassified. Fourth, DETP and DNA methylation were measured at the same baseline visit, so temporal ordering between exposure and mediator cannot be established. Causal mediation additionally requires no unmeasured confounding in the exposure–mediator, mediator–outcome, and exposure–outcome relationships; these assumptions cannot be verified in the present observational data. The mediation results should therefore be interpreted as hypothesis-generating statistical associations rather than evidence of a causal pathway. Creatinine adjustment may be biased in middle-aged and older adults because urinary creatinine depends on muscle mass, renal function, and cardiovascular health; urinary specific gravity was not measured, although sensitivity analyses using creatinine as a covariate yielded consistent results (Table 2). Diet is also a potential confounder because fruit and vegetable consumption may raise urinary organophosphate metabolites through pesticide residues while also lowering cardiovascular risk. We therefore tested sequential adjustment for poverty–income ratio, education, fruit and vegetable intake, total cholesterol, C-reactive protein, estimated glomerular filtration rate, physical activity, and cardiovascular- or diabetes-related medication use in sensitivity analyses; the DETP estimate was not materially attenuated (Table 2). Residual confounding from imperfect dietary assessment or unmeasured occupational history cannot, however, be excluded. Reverse causation is also possible if baseline cardiovascular or kidney disease alters creatinine-adjusted urinary metabolite concentrations. Multiple testing across metabolites, methylation markers, mediation models, and prediction analyses increases the possibility of false-positive findings. Although FDR correction was applied within the main testing families, the nominal indirect effects for MonoPP and ZhangAge did not remain significant after correction across the five mediation models.

The *in vitro* experiments carry additional limitations. Human exposure was assessed using urinary DETP, whereas cells were treated with chlorpyrifos, a parent compound with distinct toxicokinetics. Chlorpyrifos was applied at 10–50 μM—above typical environmental levels—in THP-1 cells for 24 h and may partly reflect nonspecific cellular stress rather than low-dose environmental exposure. THP-1 cells are an immortalized monocyte line and do not recapitulate primary vascular immune cells or chronic cardiovascular aging. We measured cytokine and DNMT1/TET2 mRNA and global 5-mC but not protein abundance, locus-specific methylation, epigenetic clocks, or functional monocyte phenotypes; global hypermethylation is therefore not equivalent to epigenetic age acceleration and does not validate the ZhangAge or HorvathAge mediation findings (41, 48).

## 5 Conclusion

In this exploratory NHANES analysis, higher urinary DETP was associated with greater heart disease mortality in older adults. MonoPP and ZhangAge showed suggestive indirect effects at raw *p* < 0.05 in separate exploratory models, with estimated proportions mediated of approximately 8–9%; however, neither effect remained statistically significant after FDR correction. Most of the excess risk therefore remained unexplained by the measured methylation markers. These findings generate hypotheses linking organophosphate exposure, DNA methylation, and heart disease mortality; they should not be interpreted as evidence of a causal exposure–methylation–mortality pathway until replicated in larger cohorts with repeated exposure assessment.

## Data Availability

The datasets presented in this study can be found in online repositories. The names of the repository/repositories and accession number(s) can be found in the article/supplementary material.

## References

[1] Jaiswal S Singh B Dhingra I Joshi A Kodgire P. Bioremediation and bioscavenging for elimination of organophosphorus threats: an approach using enzymatic advancements. Environ Res. (2024) 252:118888. doi: 10.1016/j.envres.2024.118888, 38599448

[2] Singh BK. Organophosphorus-degrading bacteria: ecology and industrial applications. Nat Rev Microbiol. (2009) 7:156–64. doi: 10.1038/nrmicro2050, 19098922

[3] Saad H Elfeky SA El-Gamel NEA Abo Dena AS. Organophosphate pesticides: a review on classification, synthesis, toxicity, remediation and analysis. RSC Adv. (2025) 15:40802–22. doi: 10.1039/d5ra05552k, 41159084 PMC12557714

[4] Aroniadou-Anderjaska V Figueiredo TH Furtado M d A Pidoplichko VI Braga MFM. Mechanisms of organophosphate toxicity and the role of acetylcholinesterase inhibition. Toxics. (2023) 11:866. doi: 10.3390/toxics11100866, 37888716 PMC10611379

[5] Fghihi-Zarandi A Dabaghzadeh F Vaziri A Karami-Mohajeri S Ghorbaninejad B Zamani A . Occupational risk assessment of organophosphates with an emphasis on psychological and oxidative stress factors. Toxicol Ind Health. (2022) 38:342–50. doi: 10.1177/07482337221096315, 35513771

[6] Samareh A Asadikaram G MojtabaAbbasi-Jorjandi n Abdollahdokht D Abolhassani M Khanjani N . Occupational exposure to pesticides in farmworkers and the oxidative markers. Toxicol Ind Health. (2022) 38:455–69. doi: 10.1177/07482337221106754 35701988

[7] Lu C Toepel K Irish R Fenske RA Barr DB Bravo R. Organic diets significantly lower children’s dietary exposure to organophosphorus pesticides. Environ Health Perspect. (2006) 114:260–3. doi: 10.1289/ehp.8418, 16451864 PMC1367841

[8] Bi R Su G. From target to unknown: comprehensive identification and risk assessment of organophosphate pesticides in foodstuffs. J Agric Food Chem. (2025) 73:14058–69. doi: 10.1021/acs.jafc.5c02513, 40407150

[9] Wang X Tian M Shen Z Tian K Fei Y Cheng Y . Comprehensive cross-sectional study of the triglyceride glucose index, organophosphate pesticide exposure, and cardiovascular diseases: a machine learning integrated approach. Toxics. (2025) 13:118. doi: 10.3390/toxics13020118, 39997933 PMC11860532

[10] Guo X Wang H Song Q Li N Liang Q Su W . Association between exposure to organophosphorus pesticides and the risk of diabetes among US adults: cross-sectional findings from the National Health and nutrition examination survey. Chemosphere. (2022) 301:134471. doi: 10.1016/j.chemosphere.2022.134471, 35367493

[11] Zhu S Zhou Y Chao M Zhang Y Cheng W Xu H . Association between organophosphorus insecticides exposure and osteoarthritis in patients with arteriosclerotic cardiovascular disease. BMC Public Health. (2024) 24:1873. doi: 10.1186/s12889-024-19414-9, 39004719 PMC11247838

[12] Hernández-Mariano JÁ Baltazar-Reyes MC Salazar-Martínez E Cupul-Uicab LA. Exposure to the pesticide DDT and risk of diabetes and hypertension: systematic review and meta-analysis of prospective studies. Int J Hyg Environ Health. (2022) 239:113865. doi: 10.1016/j.ijheh.2021.113865, 34700204

[13] Leonel Javeres MN Habib R Judith Laure N Abbas Shah ST Valis M Kuca K . Chronic exposure to organophosphates pesticides and risk of metabolic disorder in cohort from Pakistan and Cameroon. Int J Environ Res Public Health. (2021) 18:2310. doi: 10.3390/ijerph18052310, 33652791 PMC7967685

[14] Conole ELS Robertson JA Smith HM Cox SR Marioni RE. Epigenetic clocks and DNA methylation biomarkers of brain health and disease. Nat Rev Neurol. (2025) 21:411–21. doi: 10.1038/s41582-025-01105-7, 40533525

[15] Horvath S Raj K. DNA methylation-based biomarkers and the epigenetic clock theory of ageing. Nat Rev Genet. (2018) 19:371–84. doi: 10.1038/s41576-018-0004-3, 29643443

[16] Morgan RK Wang K Svoboda LK Rygiel CA Lalancette C Cavalcante R . Effects of developmental Lead and phthalate exposures on DNA methylation in adult mouse blood, brain, and liver: a focus on genomic imprinting by tissue and sex. Environ Health Perspect. (2024) 132:67003. doi: 10.1289/EHP14074, 38833407 PMC11166413

[17] Shah S McRae AF Marioni RE Harris SE Gibson J Henders AK . Genetic and environmental exposures constrain epigenetic drift over the human life course. Genome Res. (2014) 24:1725–33. doi: 10.1101/gr.176933.114, 25249537 PMC4216914

[18] Fu H Tan P Wang R Li S Liu H Yang Y . Advances in organophosphorus pesticides pollution: current status and challenges in ecotoxicological, sustainable agriculture, and degradation strategies. J Hazard Mater. (2022) 424:127494. doi: 10.1016/j.jhazmat.2021.127494, 34687999

[19] Rusiecki JA Beane Freeman LE Bonner MR Alexander M Chen L Andreotti G . High pesticide exposure events and DNA methylation among pesticide applicators in the agricultural health study. Environ Mol Mutagen. (2017) 58:19–29. doi: 10.1002/em.22067, 27996157 PMC5416937

[20] Schaffner SL Casazza W Artaud F Konwar C Merrill SM Domenighetti C . Genetic variation and pesticide exposure influence blood DNA methylation signatures in females with early-stage Parkinson’s disease. npj Park Dis. (2024) 10:98. doi: 10.1038/s41531-024-00704-3, 38714693 PMC11076573

[21] Johnson CL Paulose-Ram R Ogden CL Carroll MD Kruszon-Moran D Dohrmann SM . National health and nutrition examination survey: analytic guidelines, 1999-2010. Vital Health Stat 2. (2013):1–24. 25090154

[22] NHANES - National Health and Nutrition Examination Survey Homepage. Available online at: https://www.cdc.gov/nchs/nhanes/index.htm (Accessed January 1, 2026).

[23] WHO (2015) International statistical classification of diseases and related health problems. Available online at: https://iris.who.int/handle/10665/246208 (Accessed January 1, 2026)

[24] O’Brien KM Upson K Buckley JP. Lipid and creatinine adjustment to evaluate health effects of environmental exposures. Curr Environ Health Rep. (2017) 4:44–50. doi: 10.1007/s40572-017-0122-7, 28097619 PMC5323273

[25] Salas LA Koestler DC Butler RA Hansen HM Wiencke JK Kelsey KT . An optimized library for reference-based deconvolution of whole-blood biospecimens assayed using the Illumina HumanMethylationEPIC BeadArray. Genome Biol. (2018) 19:64. doi: 10.1186/s13059-018-1448-7, 29843789 PMC5975716

[26] Lu AT Seeboth A Tsai P-C Sun D Quach A Reiner AP . DNA methylation-based estimator of telomere length. Aging. (2019) 11:5895–923. doi: 10.18632/aging.102173, 31422385 PMC6738410

[27] Vidal-Bralo L Lopez-Golan Y Gonzalez A. Simplified assay for epigenetic age estimation in whole blood of adults. Front Genet. (2016) 7:126. doi: 10.3389/fgene.2016.00126, 27471517 PMC4943959

[28] Zhang Q Vallerga CL Walker RM Lin T Henders AK Montgomery GW . Improved precision of epigenetic clock estimates across tissues and its implication for biological ageing. Genome Med. (2019) 11:54. doi: 10.1186/s13073-019-0667-1, 31443728 PMC6708158

[29] Horvath S. DNA methylation age of human tissues and cell types. Genome Biol. (2013) 14:R115. doi: 10.1186/gb-2013-14-10-r115, 24138928 PMC4015143

[30] Hannum G Guinney J Zhao L Zhang L Hughes G Sadda S . Genome-wide methylation profiles reveal quantitative views of human aging rates. Mol Cell. (2013) 49:359–67. doi: 10.1016/j.molcel.2012.10.016, 23177740 PMC3780611

[31] Horvath S Oshima J Martin GM Lu AT Quach A Cohen H . Epigenetic clock for skin and blood cells applied to Hutchinson Gilford progeria syndrome and ex vivo studies. Aging. (2018) 10:1758–75. doi: 10.18632/aging.101508, 30048243 PMC6075434

[32] Lin Q Weidner CI Costa IG Marioni RE Ferreira MRP Deary IJ . DNA methylation levels at individual age-associated CpG sites can be indicative for life expectancy. Aging. (2016) 8:394–401. doi: 10.18632/aging.100908, 26928272 PMC4789590

[33] Weidner CI Lin Q Koch CM Eisele L Beier F Ziegler P . Aging of blood can be tracked by DNA methylation changes at just three CpG sites. Genome Biol. (2014) 15:R24. doi: 10.1186/gb-2014-15-2-r24, 24490752 PMC4053864

[34] Belsky DW Caspi A Arseneault L Baccarelli A Corcoran DL Gao X . Quantification of the pace of biological aging in humans through a blood test, the DunedinPoAm DNA methylation algorithm. eLife. (2020) 9:e54870. doi: 10.7554/eLife.54870, 32367804 PMC7282814

[35] Lu AT Quach A Wilson JG Reiner AP Aviv A Raj K . DNA methylation GrimAge strongly predicts lifespan and healthspan. Aging. (2019) 11:303–27. doi: 10.18632/aging.101684, 30669119 PMC6366976

[36] Li Q Lesseur C Srirangam P Kaur K Hermetz K Caudle WM . Associations between prenatal organophosphate pesticide exposure and placental gene networks. Environ Res. (2023) 224:115490. doi: 10.1016/j.envres.2023.115490, 36828252 PMC10054353

[37] Timchalk C Busby A Campbell JA Needham LL Barr DB. Comparative pharmacokinetics of the organophosphorus insecticide chlorpyrifos and its major metabolites diethylphosphate, diethylthiophosphate and 3,5,6-trichloro-2-pyridinol in the rat. Toxicology. (2007) 237:145–57. doi: 10.1016/j.tox.2007.05.007, 17590257

[38] Holme F Thompson B Holte S Vigoren EM Espinoza N Ulrich A . The role of diet in children’s exposure to organophosphate pesticides. Environ Res. (2016) 147:133–40. doi: 10.1016/j.envres.2016.02.003, 26870919 PMC4821762

[39] Medrano-Bosch M Simón-Codina B Jiménez W Edelman ER Melgar-Lesmes P. Monocyte-endothelial cell interactions in vascular and tissue remodeling. Front Immunol. (2023) 14:1196033. doi: 10.3389/fimmu.2023.1196033, 37483594 PMC10360188

[40] Jaipersad AS Lip GYH Silverman S Shantsila E. The role of monocytes in angiogenesis and atherosclerosis. J Am Coll Cardiol. (2014) 63:1–11. doi: 10.1016/j.jacc.2013.09.019, 24140662

[41] Han Y Liu S Ochieng BO Gu Y Kong L Wang G . Multiscale mechanical study of Proanthocyanidins for recovering residual stress in Decellularized blood vessels. Adv Healthc Mater. (2025) 14:e2402250. doi: 10.1002/adhm.202402250, 39801220

[42] Xu S Kamato D Little PJ Nakagawa S Pelisek J Jin ZG. Targeting epigenetics and non-coding RNAs in atherosclerosis: from mechanisms to therapeutics. Pharmacol Ther. (2019) 196:15–43. doi: 10.1016/j.pharmthera.2018.11.003, 30439455 PMC6450782

[43] Dopheide JF Obst V Doppler C Radmacher M-C Scheer M Radsak MP . Phenotypic characterisation of pro-inflammatory monocytes and dendritic cells in peripheral arterial disease. Thromb Haemost. (2012) 108:1198–207. doi: 10.1160/TH12-05-0327, 23093299

[44] Wang C Zhong G Liu C Hong S Guan X Xiao Y . DNA methylation aging signatures of multiple metals exposure and their mediation effects in metal-associated mortality: evidence from the Dongfeng-Tongji cohort study. J Hazard Mater. (2024) 465:133200. doi: 10.1016/j.jhazmat.2023.133200, 38113735

[45] Zou Y Huang J Tang X Xu J. Associations between five indicators of epigenetic age acceleration and all-cause and cause-specific mortality among US adults aged 50 years and older. Clin Epigenetics. (2025) 17:66. doi: 10.1186/s13148-025-01872-6, 40301953 PMC12038942

[46] Chen X-L Zhao Q-Q Lin S-R He X-L Zhang X-J Li S-J . Epigenetic clocks as mediators of health behaviors and mortality in middle-aged and older adults. J Nutr Health Aging. (2025) 29:100602. doi: 10.1016/j.jnha.2025.100602, 40505553 PMC12276600

[47] Jiang C Xie N Sun T Ma W Zhang B Li W. Xanthohumol inhibits TGF-β1-induced cardiac fibroblasts activation via mediating PTEN/Akt/mTOR signaling pathway. Drug Des Devel Ther. (2020) 14:5431–9. doi: 10.2147/DDDT.S282206, 33324040 PMC7732164

[48] Ham J Koh J Kim J Cho J-Y Kim T Chung DH . Modulating the PD-1-FABP5 axis in ILC2s to regulate adipose tissue metabolism in obesity. Mol Ther. (2025) 33:1842–59. doi: 10.1016/j.ymthe.2025.02.015, 39949060 PMC11997476

[49] Chen Y Yim WY Li C Wang Z Hou J Peng Y . T cell senescence induced by DNMT1 deletion mediates cardiac transplant tolerance via altered CDKN1A methylation. Mol Ther. (2026) 34:3484–502. doi: 10.1016/j.ymthe.2026.02.015, 41691367 PMC13239813

[50] Wang H Zhou L. Random survival forest with space extensions for censored data. Artif Intell Med. (2017) 79:52–61. doi: 10.1016/j.artmed.2017.06.005, 28641924

[51] Zhao J-J Zhang J Li S Wang Q Mo Q Yu H. Dietary iron attenuates epigenetic aging through DNA methylation remodeling and extends survival in older adults. Clin Epigenetics. (2025) 17:181. doi: 10.1186/s13148-025-01986-x, 41163063 PMC12573944

[52] Levine ME Lu AT Quach A Chen BH Assimes TL Bandinelli S . An epigenetic biomarker of aging for lifespan and healthspan. Aging. (2018) 10:573–91. doi: 10.18632/aging.101414, 29676998 PMC5940111

[53] Giuliani C Biggs D Nguyen TT Marasco E De Fanti S Garagnani P . First evidence of association between past environmental exposure to dioxin and DNA methylation of CYP1A1 and IGF2 genes in present day Vietnamese population. Environ Pollut. (2018) 242:976–85. doi: 10.1016/j.envpol.2018.07.015, 30373043

[54] Dong Z Zhu Y Che R Chen T Liang J Xia M . Unraveling the complexity of organophosphorus pesticides: ecological risks, biochemical pathways and the promise of machine learning. Sci Total Environ. (2025) 974:179206. doi: 10.1016/j.scitotenv.2025.179206, 40154081

